# Perceptions of information and participation in child and adolescent mental health care: a comparison of patient and caregiver reports across inpatient and day hospital settings

**DOI:** 10.1186/s13034-026-01096-3

**Published:** 2026-05-17

**Authors:** Daria Nolkemper, Dirk Lubbe, Ferdinand Keller, Bettina K. Doering, Stefanie Bienioschek

**Affiliations:** 1 Brandenburg Medical School Theodor Fontane (MHB), Neuruppin, Germany; 2 Department of Psychiatry, Psychotherapy und Psychosomatics of Children and Adolescents, University Hospital Ruppin-Brandenburg, Neuruppin, Germany; 3 Department of Child and Adolescent Psychiatry, Psychosomatics and Psychotherapy, Ulm University Hospital, Ulm, Germany; 4 Department of Psychology, Kiel University, Kiel, Germany; 5 Faculty of Health Sciences, The Brandenburg Medical School and the Brandenburg University of Technology Cottbus, University of Potsdam, Potsdam, Neuruppin, Cottbus/Senftenberg, Rüdersdorf, Germany

**Keywords:** Child and adolescent psychiatry, Inpatient treatment, Day hospital treatment, Person-centered care, Participation, Information, Treatment satisfaction

## Abstract

**Background:**

In child and adolescent mental health services (CAMHS), the principles of information provision and participatory decision-making have gained prominence, reflecting both legal mandates and ethical standards. Within the last few years, the number of day hospital treatment programs has increased and political decisions led to a reduction in inpatient beds. Given the structural and procedural differences between the treatment settings, this study examines whether patients’ and caregivers’ ratings of information and participation differ between an inpatient treatment and a day hospital treatment.

**Method:**

Patients and caregivers answered the “Broad Evaluation of Satisfaction with Treatment” (BEST) questionnaire before discharge from inpatient or day hospital treatment. Two exploratory new subscales were pragmatically composed from existing items in the BEST: An information and a participation subscale. Data from 127 children, 93 caregivers, and 136 adolescents regarding their perception of information provision and participation were analyzed to examine potential differences between the settings.

**Results:**

Children and caregivers rated information provision and participation equally high in both treatment settings. Adolescents, however, reported higher satisfaction with information provision during day hospital treatment compared to inpatient treatment. Gender and diagnosis affected the evaluations of participation within the adolescent sample. In the child sample, gender had an effect on the ratings of participation. The subscales Information and Participation show good reliabilities in the adolescent and in the caregiver samples, whereas within the child sample, only the participation subscale shows sufficiently good psychometric properties.

**Conclusion:**

Despite structural and conceptual differences between the settings, there are no significant differences in the perception of participation. Adolescents in the day hospital, however, reported higher satisfaction with information than adolescents in inpatient treatment. One possible explanation could be a participatory treatment planning tool that is used in the day hospital for adolescents. This tool addresses the item “Goals of treatment discussed” which is located in the information subscale. The subscales of the BEST questionnaire, particularly those related to information and participation, may be helpful in highlighting and fostering person-centered care, especially for adolescents.

**Supplementary Information:**

The online version contains supplementary material available at 10.1186/s13034-026-01096-3.

## Background

Participation is increasingly considered a key quality principle in health promotion [1, 2]. There are various definitions of the term. The model by Wright et al. [3] considers co-determination, partial decision-making authority and single decision-making authority as participation. Information, consultation and engagement are preliminary stages that lead to participation [3]. According to a meta-analysis on factors that facilitate adolescents’ participation in mental health treatment, receiving “sufficient, understandable and age-appropriate information” [4], p.12] helps building a collaborative relationship which positively affects the patients’ motivation and involvement in their treatment [4].

Participation has a positive impact on children’s mental health, particularly through an enhanced sense of self-efficacy, which leads to an improvement in self-worth [5]. It can therefore be argued that participation is important for children’s personal development. In the context of child and adolescent psychiatric treatment, more participation is associated with higher treatment satisfaction [6–8]. Additionally, shared decision making (SDM), i.e. participation, can increase treatment adherence and the understanding of the disease [9].

Participation is a legal right for children. With the ratification of the Convention on the Rights of the Child (CRC), countries have committed themselves to prioritizing the welfare of children in both private and public institutions. Article 12 of the Convention affirms children’s right to express their opinions and to have those opinions considered, in accordance with their age and maturity [10]. Complementarily, the European Association for Children in Hospital (EACH) Charter specifically addresses the rights of children in medical settings, emphasizing their right to be informed and actively involved in health care decisions affecting their lives and health [9]. The growing emphasis on information and participation in medical treatment is also reflected in recent developments in German legislature (see Patient Rights Act of 2013).

Evaluation is an essential aspect of participation, as it enables patients to voice their opinions and potentially catalyze changes [11]. One measurement tool for children’s and adolescents’ psychiatric and psychotherapeutic treatment in Germany is the “Broad Evaluation of Satisfaction with Treatment” (BEST) questionnaire, which assesses the following aspects of patient satisfaction: therapeutic relationship, treatment environment, general satisfaction and treatment success [12]. Evaluations using this questionnaire since 2011 have shown an increase in satisfaction with some aspects of information and participation, such as receiving information on coercive measures and collaboratively setting treatment goals [13]. However, recent studies also indicate that patients still experience less participation than they desire [8, 14]. A scoping review from 2020 identified three key barriers to implementing participation in CAMHS: (1) staff reluctance, (2) patient reluctance, and (3) structural barriers [15]. There has been research examining the first two barriers recently. Two studies highlight a strong demand for participation among patients [8, 14]. Another study surveyed mental health professionals and actually discovered a positive attitude towards participation among staff [16]. To the best of our knowledge, there is no research yet concerning the question whether the information and participation of patients or caregivers are influenced by structural barriers. Therefore, our goal was to take a closer look at the perception of information and participation within different treatment settings, since they operate with different structural and procedural frameworks.

The current political landscape in Germany is shaped by various challenges, including a reform of the healthcare system, particularly hospital care [17]. In CAMHS, the reform focuses on increasing outpatient and day hospital capacities to enhance access to home-based care [18]. In recent years, day hospitals have been established, with outpatient care gaining greater importance [19]. Patients with severe mental health conditions, however, may require hospitalization in either a day hospital or an inpatient ward. Currently, nearly half of all child and adolescent psychiatric treatments in Germany are provided in day hospitals [18]. Day hospital treatment and inpatient treatment settings differ fundamentally in structure: day hospital patients attend weekdays until afternoon, returning home evenings, nights and weekends. In day hospital treatment, patients gradually reintegrate into school over approximately three weeks before discharge, depending on individual needs. Day hospitals are often closer to home and school, allowing patients to stay connected to their social environment [20]. To support families in accessing care, the health insurance covers transportation costs to the day hospital, if caregivers are unable to organize transport themselves. Almost all admissions to the day hospital are planned in advance. Patients who arrive in a crisis are admitted as inpatients. Inpatient wards are divided in acute wards and therapeutic sections, or may combine both. Acute care wards primarily treat emergency patients experiencing acute crises, while therapeutic wards treat patients who have been admitted on a regular basis. Following an initial familiarization period of approximately two weeks, patients may be allowed an overnight leave, in accordance with therapeutic objectives and contingent on adequate psychological stability and a sufficiently stable social environment. In contrast to day hospitals, the gradual reintegration process from inpatient treatment to the home environment is often difficult to realize due to the long distances between facilities and the patients’ homes, among other things. In Germany, CAMHS frequently serve areas that are three times larger than those for adult patients [17, 21].

Day hospital units seem to be more cost-effective [22], but studies comparing the effectiveness of different treatment settings are limited and do not provide a clear picture [20]. Existing research shows that both treatment settings reduce symptoms and improve functional outcomes and quality of life, with no significant differences in treatment satisfaction [22, 23]. The caregivers’ participation can play a key role in treatment success across both settings, since caregivers can help reinforce the effects of therapy in everyday life [24]. Some interventions even require the participation of the patient’s caregivers [25]. According to clinicians, involving parents in treatment is beneficial, particularly in cases of externalizing disorders [26]. Additionally, the success of patient participation appears to be influenced by the caregivers’ participation [27], as studies show that family support increases participation [4] and positively affects person-centered care [28]. A study investigating the relationship between shared decision making (SDM) and treatment outcome found that the strongest effect occurred when both parents and children experienced higher levels of SDM [29]. A study investigating SDM with younger children (< 7 years) and their parents found strong associations between higher levels of SDM and treatment satisfaction [30].

Information and participation of children and their caregivers can contribute to treatment success. However, perceptions of patients and caregivers may differ [31, 32], highlighting the need for a dual-perspective assessment. Previous research has mainly focused on older children; for example, despite the availability of a version of the BEST questionnaire for younger children in the manual, no studies using this tool have been published for this group to date [33]. To the best of our knowledge, no studies have yet explored the impact of the treatment setting (day hospital vs. inpatient treatment) on the perception of information and participation among children, adolescents, and caregivers. Therefore, the aim of the current study is to examine whether the treatment setting influences how children, adolescents and caregivers perceive being informed and included in the treatment process.

## Methods

### Treatment settings

The Department of Child and Adolescent Psychiatry and Psychotherapy at the University Hospital Ruppin–Brandenburg comprises two different settings, i.e., inpatient treatment and day hospital treatment. Inpatient care is provided in three wards organized by developmental stage and covering both emergency and standard care.

Both settings are open to children and adolescents. In both settings before or during admission, patients and their caregivers are offered a child-friendly informational brochure, available in both paper format and as a downloadable version from the hospital´s website. Originally developed by Ulm University Hospital, the brochure has been adapted to reflect local contextual factors [21, 34]. In inpatient wards and day hospitals, treatment is delivered by a multidisciplinary team, led by a specialist in child and adolescent psychiatry and psychotherapy. It includes nursing and educational support, individual and group psychotherapy, pharmacotherapy, specialized therapies such as occupational and art therapy, and social services. Caregivers regularly participate in both settings, with the type of involvement—such as family counseling sessions, observation of interactions, or participation in additional group programs—adapted to the specific therapeutic needs.

There are two main differences between the settings. Unlike the inpatient setting, the day hospital offers biweekly multifamily therapy sessions with the specific topics varying based on the needs of the patients and their families [35]. In contrast to the inpatient setting and the day hospital setting for children, the day hospital for adolescents uses a participatory and patient-oriented treatment planning approach. This approach employs a traffic light system, in which green indicates areas that are going well, yellow highlights aspects that need attention, and red signals areas that have a greater need of improvement. It is implemented after the diagnostic phase, based on the frameworks established by Kölch et al. [36] and Möhrle et al. [37].

### Procedure

Data collection took place from 2021 until 2025 within the routine service evaluation of the hospital. The study was approved by the ethical committee of the University Hospital Ruppin-Brandenburg (positive vote: E-03-20230224). Prior to discharge, children, adolescents and caregivers were invited to complete the BEST questionnaire [33]. In addition to the BEST, medical and psychotherapeutic staff in charge of cases and graduate psychotherapy students recorded the main category of the diagnosis according to ICD-10 (externalizing disorder: F1, F60–62, F63, F90/91; internalizing disorder: F32–34, F40/41, F42, F50; neither: F2, F30/31, F43-45, F8, F9). If patients presented with comorbidities, the staff determined which diagnosis was primary based on the patient’s clinical presentation. They also recorded the psychosocial functioning level (Axis VI of the multiaxial classification) [38], treatment duration (more than two weeks and less than two weeks); hospital admission via legal decision, regular admission and admission as emergency (i.e., crisis), setting (day hospital vs. inpatient ward), and patient gender.

### Instrument

The BEST questionnaires consist of 20 items (questionnaire for children; BEST-C), 27 items (questionnaire for adolescents; BEST-A) or 22 items (questionnaire for parents and caregivers; BEST-P). The questionnaire for children is generally used with children up to and including the age of 12 years, with no minimum age requirement specified [33], the BEST for adolescents is used from the age of 13 years onwards. In our sample, the BEST-C was used in the children’s wards and the BEST-A was used in the adolescents’ wards, with patients assigned to the wards based on their developmental stage (i.e., not age in years). The BEST items assess patient satisfaction and are rated from 1 (strongly disagree) to 5 (strongly agree). These items can be combined to a total score. In addition, there are three subscales, i.e., (1) therapeutic relationship, (2) environment, and (3) general satisfaction and treatment success; however, the latter is not available for children [12]. Scores of all subscales are calculated as average item ratings between 1 and 5, with higher ratings reflecting a more positive assessment. The questionnaire includes additional items asking for complimentary information, e.g., adequacy of treatment duration, and three open-ended questions [33]. The BEST for adolescents and for caregivers demonstrated convergent validity by high correlations with the treatment evaluation questionnaires (FBB – Mattejat and Remschmidt 1998; see [33]). The convergent validity of the BEST for children has not been demonstrated due to a lack of a respective second questionnaire in German for this age group. It can be assumed that the BEST questionnaires have high content validity, as the instrument was developed in a clinical setting (through expert and patient surveys in focus groups).

The BEST questionnaire includes multiple items specifically addressing information and participation. To examine patients’ perceptions of these domains in greater detail, two subscales were constructed from relevant items: an information and a participation subscale. Because the study was based on routinely collected clinical data, the available items were pragmatically selected based on their conceptual fit with the constructs of interest. Specifically, the information subscale contains items about information on different topics like medication, coercive measures, discharge time and being included in setting treatment goals and the participation subscale contains items about trust and co–determination. The information and participation subscales were constructed for exploratory purposes and have not been formally validated. This procedure was intended as a pragmatic strategy to capture the constructs of interest within the constraints of the available routine data rather than as a psychometric scale development.

**Fig. 1:**
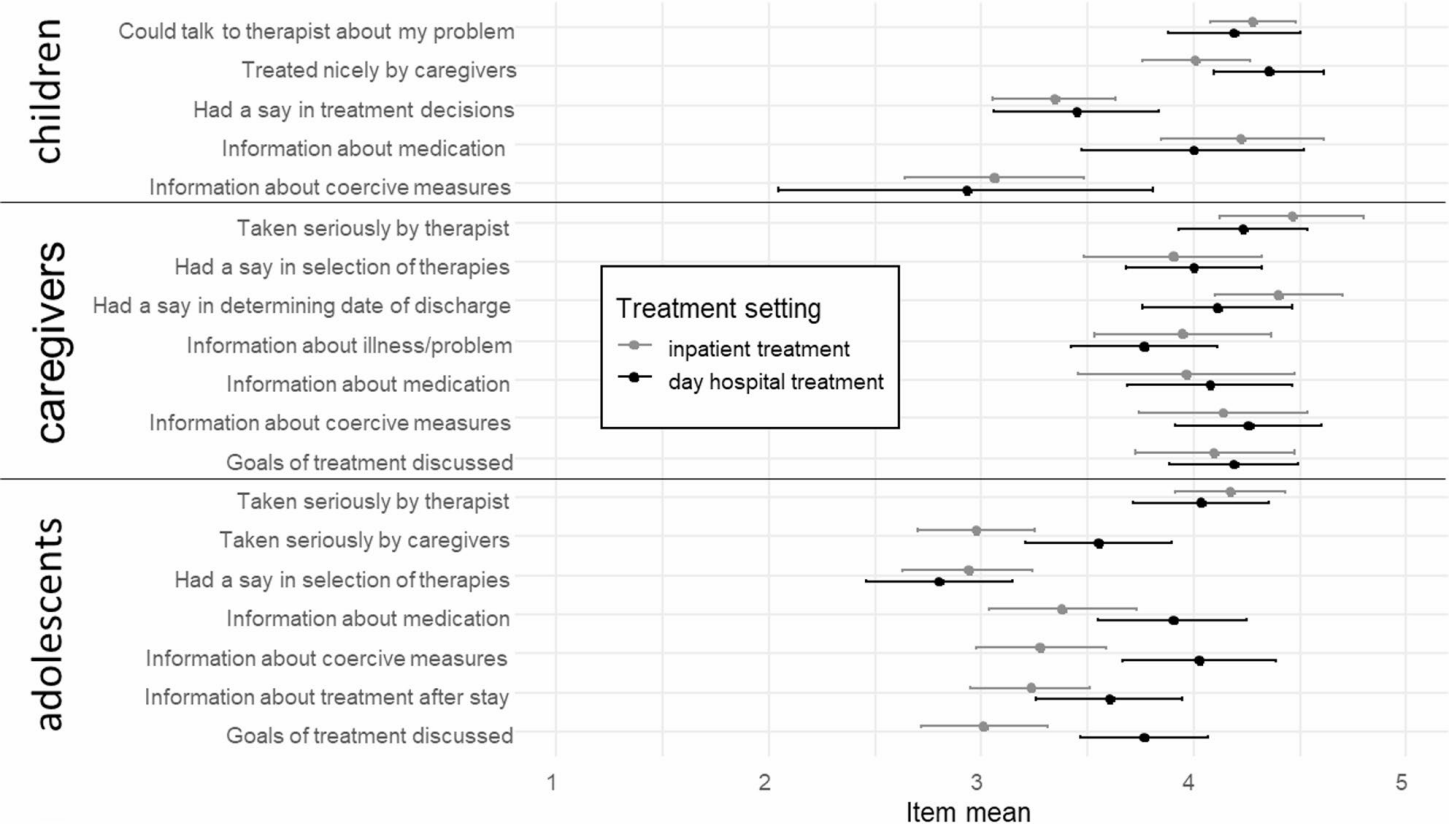
Means and 95% confidence intervals of the BEST items pertaining to the information and participation subscales by sample and treatment setting

For a content of the items see Fig. 1. Table 5 provides the original item numbers in the BEST-C, BEST-P and BEST-A and descriptive statistics. The reliability estimates (Cronbach’s Alpha) are displayed in Table 4.

### Participants

A total of 148 children, 117 caregivers (71 caregivers of children and 22 caregivers of adolescents) and 195 adolescents completed the survey. Importantly, they represent distinct samples, as anonymity prevents a matching of minor-caregiver dyads. This was a convenience sample from routine outcome monitoring. The final analytic sample was obtained through a two-step case selection process. In the first step, cases with three or more missing responses on the BEST (with the exception of items that are not applicable to all patients, e.g., items on medication information for patients who did not receive medication) were excluded. This step resulted in the exclusion of five cases from the child sample and fourteen cases from the adolescent sample.

In the second step, cases classified as gender diverse were removed due to the insufficient number of such cases for meaningful significance testing. In addition, cases with no recorded diagnosis were excluded because assignment to diagnostic group was required for the planned analyses. Specifically, one case identified as gender diverse was removed from the child and caregiver samples, and five cases were removed from the adolescent sample. After the removal of cases without a recorded diagnosis, the final sample sizes are *n* = 127 (85.5% of initial records) for the child sample, *n* = 93 (79.5% of initial records) for the caregiver sample, and *n* = 136 (69.7% of initial records) for the adolescent sample.

### Statistical analysis

Data analyses were performed using R (version 4.4.1 [39]). For each of the three datasets, we calculated descriptive statistics (means with 95% confidence intervals and standard deviations) and reliability estimates for the individual BEST scales, as well as for the two newly assembled participation and information subscales. Even though the two new subscales were constructed for exploratory purposes, we also computed item means and item-discrimination values for all questionnaire items that contributed to their calculation. For comparison and validation, we also calculated reliabilities of the new subscales based on the manual samples of the BEST questionnaire [12, 33]. The manual samples consist of 852 children, 1582 adolescents and 1998 caregivers from seven Departments of Child and Adolescent Psychiatry and Psychotherapy across various federal states in Germany.

Analysis of variance (ANOVA) was used to examine the effects of patient gender (male, female^1^), treatment setting (inpatient vs. day hospital), and category of diagnosis (externalizing, internalizing or neither) on information and participation scores. Specifically, separate ANOVAs were conducted for the two subscales across all three datasets. In a stepwise procedure, any interaction effects that did not significantly contribute (at the 5% significance level) to explaining the dependent variable were removed.

We acknowledge that repeated testing increases the overall probability of Type I errors. However, these analyses are intended as descriptive explorations of potentially influential variables, rather than tests of a priori specified hypotheses. Thus, the findings are exploratory rather than confirmatory and test statistics at conventional significance levels should be interpreted as descriptive indicators rather than confirmatory tests.

## Results

### Descriptive statistics

#### Participant characteristics

Tables 1, 2, and 3 contain the descriptive statistics of the three samples. Descriptive statistics are given for the entire samples and separately for each setting (inpatient and day hospital). The plurality of the children was male (71.7%) and diagnosed with an externalizing disorder (50.4%). Based on the sociodemographic data completed by healthcare personnel, 1.6% of the children in the sample were admitted to psychiatric care by legal mandate. Of the caregivers, 55.9% reported that their child was male and 45.2% indicated that their child had been diagnosed with an externalizing disorder. The caregiver sample consists of 22 caregivers of adolescents and 71 caregivers of children. For 5.4% of the cases in the caregiver sample, health care personnel reported that the respondent’s child or adolescent were in psychiatric care by legal mandate. Among the adolescents, 11.0% presented with an externalizing disorder and 64.7% with an internalizing disorder. Being in psychiatric care by legal mandate was recorded for 6.6% of the cases.

**Table 1:** Descriptive statistics of the child sample

Age range	6–14 years	All cases (*n* = 127)		Inpatient treatment (*n* = 79)		Day hospital treatment (*n* = 48)	
		Mean	SD	Mean	SD	Mean	SD
		9.57	1.94	9.90	1.92	9.27	1.92
		*n*	%	*n*	%	*n*	%
Gender	Male	91	71.7	57	72.2	34	70.8
	Female	36	28.3	22	27.8	14	29.2
Diagnosis	Externalizing	64	50.4	33	41.8	31	64.6
	Internalizing	26	20.5	25	31.6	1	2.1
	Neither	37	29.1	21	26.6	16	33.3
Order ^a^	No	121	95.3	77	97.5	44	91.7
	Yes	2	1.6	1	1.3	1	2.1
Duration ^b^	>= 2w	113	89.0	65	82.3	48	100.0
	< 2w	8	6.3	8	10.1	0	0.0
Crisis ^a^	No	83	65.4	36	45.6	47	97.9
	Yes	40	31.5	39	49.4	1	2.1

%: percentages of the total number of cases. Order: admitted via court decision. ^a^ 4 missing cases; ^b^ 6 missing cases

**Table 2:** Descriptive statistics of the children and adolescents’ characteristics in the caregiver sample

		All cases (*n* = 93)		Inpatient treatment (*n* = 52)		Day hospital treatment (*n* = 41)	
		*n*	%	*n*	%	*n*	%
Patients‘ gender	Male	52	55.9	22	53.7	30	57.7
	Female	41	44.1	19	46.3	22	42.3
Patients‘ diagnosis	Externalizing	42	45.2	15	36.6	27	51.9
	Internalizing	25	26.9	15	36.6	10	19.2
	Neither	26	28.0	11	26.8	15	28.8
Order ^a^	No	83	89.2	34	82.9	49	94.2
	Yes	5	5.4	4	9.8	1	1.9
Duration ^b^	> =2w	86	92.5	34	82.9	52	100.0
	< 2w	1	1.1	1	2.4	0	0.0
Crisis ^c^	No	68	73.1	20	48.8	48	92.3
	Yes	22	23.7	21	51.2	1	1.9
		Mean	SD	Mean	SD	Mean	SD
Age		9.97	2.52	11.32	2.81	9.15	1.93

%: percentages of the total number of cases. Order: Admitted via court decision. ^a^ 5 missing cases; ^b^ 6 missing cases; ^c^ 3 missing cases

**Table 3:** Descriptive statistics of the adolescent sample

		All cases (*n* = 136)		Inpatient treatment (*n* = 80)		Day hospital treatment (*n* = 56)	
		*n*	%	*n*	%	*n*	%
Gender	Male	32	23.5	20	25.0	12	21.4
	Female	104	76.5	60	75.0	44	78.6
Diagnosis	Externalizing	15	11.0	11	13.8	4	7.1
	Internalizing	88	64.7	45	56.2	43	76.8
	Neither	33	24.3	24	30.0	9	16.1
Order ^a^	No	101	74.3	59	73.8	42	75.0
	Yes	9	6.6	7	8.8	2	3.6
Duration ^b^	>= 2w	122	89.7	68	85.0	54	96.4
	< 2w	3	2.2	3	3.8	0	0.0
Crisis ^c^	No	82	60.3	41	51.2	41	73.2
	Yes	37	27.2	31	38.8	6	10.7
		Mean	SD	Mean	SD	Mean	SD
Age		14.53	1.58	14.22	1.50	14.47	1.85

%: percentages of the total number of cases. Order: Admitted via court decision. ^a^ 26 missing cases; ^b^ 11 missing cases; ^c^ 17 missing cases

Considering the subscales Information and Participation descriptively, the means of all scores were lowest in the adolescent sample and highest in the caregiver sample (see Table 4). Internal consistencies of the newly assembled subscales, which are based on a small number of items, were acceptable in the caregiver and adolescent sample; furthermore, they were in close agreement with the values for the sample published in the BEST manual. In the child sample, the corresponding reliability estimates were comparably poor (see Table 4). However, given the small number of items, the young age of respondents, and that scale construction aimed to ensure content validity rather than a perfectly homogeneous set of items, we consider the reliability sufficient for mean analyses. It has to be kept in mind that the subscales were created pragmatically for exploratory purpose.

While for the child and caregiver samples the subscale means did not differ strongly depending on treatment setting, adolescents in day hospital treatment rated the information subscale more positively. The means for all samples and the reliabilities for the original scales of the BEST are displayed in the additional file 1.

**Table 4:** Descriptive statistics and reliability for the subscales information and participation

Sample	Subscale	#Item	Cronbach’s Alpha		Treatment setting	n	Mean	SD	95% CI	
			Current sample	Manual data					Lower	Upper
Children	Info	2	0.46	0.28	All	101	3.63	1.46	3.35	3.92
					Inpatient	72	3.59	1.47	3.25	3.93
					Day hospital	29	3.74	1.44	3.22	4.27
	Part	3	0.51	0.49	all	127	3.92	0.81	3.78	4.06
					Inpatient	79	3.88	0.81	3.70	4.06
					Day hospital	48	3.99	0.80	3.76	4.22
Caregivers	Info	4	0.78	0.78	All	93	4.05	0.96	3.85	4.24
					Inpatient	41	4.04	0.95	3.75	4.34
					Day hospital	52	4.05	0.98	3.78	4.32
	Part	3	0.78	0.77	All	93	4.18	0.99	3.98	4.38
					Inpatient	41	4.26	0.89	3.98	4.53
					Day hospital	52	4.12	1.06	3.83	4.40
Adolescents	Info	4	0.70	0.70	All	136	3.41	1.00	3.24	3.58
					Inpatient	80	3.37	0.99	3.15	3.59
					Day hospital	56	3.46	1.01	3.20	3.73
	Part	3	0.67	0.65	All	136	3.46	0.98	3.30	3.62
					Inpatient	80	3.22	0.98	3.00	3.43
					Day hospital	56	3.81	0.88	3.58	4.04

#item: number of item included in each subscale. Info= information subscale, Part = participation subscale

For more detail with respect to the contribution of single items to the information and participation subscales, Table 5 gives the corresponding item statistics.

**Table 5:** Item statistics for the information and participation measures for all three samples

Sample	Subscale	Item no.	*n*	Disc.	Mean	SD	Q1	Median	Q3
Children	Info	8	83	0.30	4.16	1.44	4.0	5.0	5.0
		24	63	0.30	3.03	1.54	1.0	3.0	4.0
	Part	10	126	0.26	4.25	0.98	4.0	5.0	5.0
		13	127	0.36	4.14	1.07	4.0	5.0	5.0
		17	126	0.36	3.38	1.33	3.0	4.0	4.0
Caregivers	Info	4	92	0.58	3.85	1.30	3.0	4.0	5.0
		5	68	0.63	4.03	1.30	3.0	5.0	5.0
		12	86	0.59	4.21	1.23	4.0	5.0	5.0
		21	92	0.57	4.15	1.15	3.8	5.0	5.0
	Part	6	93	0.74	4.33	1.12	4.0	5.0	5.0
		10	93	0.56	3.96	1.26	3.0	4.0	5.0
		17	92	0.59	4.24	1.17	4.0	5.0	5.0
Adolescents	Info	5	101	0.36	3.59	1.30	3.0	4.0	5.0
		12	114	0.57	3.54	1.34	2.0	4.0	5.0
		21	128	0.49	3.38	1.26	2.0	3.0	4.2
		25	136	0.54	3.32	1.33	2.0	3.0	4.2
	Part	6	136	0.51	4.12	1.19	4.0	5.0	5.0
		9	136	0.45	3.21	1.32	2.0	3.0	4.0
		19	135	0.48	2.88	1.35	2.0	3.0	4.0

Item no.= item number in the BEST-C, BEST-P, and BEST-A, respectively; Disc.: part-whole corrected item discrimination; Q1: 25% quantile; Q3: 75% quantile. Info= information subscale, Part = participation subscale

Figure 1 illustrates the means including their 95% confidence intervals of the items that are contained in the information and participation subscales for the individual samples separated by treatment setting. The range of the item information about coercive measures in the day hospital sample of the children was high. All items in the adolescent information subscale were rated descriptively more positive in day hospital than in inpatient treatment, which corresponds with the overall trend observed in connection with the total scores of the BEST subscales.

### Analyses of variance

The ANOVA tests for the information and participation scores for the child sample are contained in Table 6. Interaction terms are not contained in the analyses, as they were removed for lack of statistical significance. Overall, only gender had a notable effect on participation. Specifically, girls rated participation slightly higher than boys. Gender had no notable effect on information.

**Table 6:** ANOVA tests for the subscales information and participation in the child sample

Dependent variable	Effect	$\:\mathrm{d}{\mathrm{f}}_{num}$	$\:\mathrm{d}{\mathrm{f}}_{den}$	F	$\:{\eta\:}_{ges}$	*p*
Information	Treat. Setting	2	96	0.01	< 0.001	0.911
	Gender	1	96	6.44	0.063	0.013
	Diagnosis	1	96	1.22	0.025	0.301
Participation	Treat. Setting	1	122	0.03	< 0.001	0.861
	Gender	1	122	5.00	0.039	0.027
	Diagnosis	2	122	0.84	0.014	0.434

Treat. Setting = Treatment setting: inpatient, day hospital; gender: female, male; diagnosis: externalizing, internalizing, neither; $\:\mathrm{d}\mathrm{f}$ = degrees of freedom; $\:{\eta\:}_{ges}$ = explained variance (generalized eta squared)

Table 7 contains the ANOVA tests for the caregiver sample. Neither interactions nor main effects reached statistical significance for information and participation. Caregiver findings should be interpreted cautiously due to heterogeneity in the sample that arises from combining parents of child and adolescent cases and unequal distribution across treatment settings.

**Table 7:** ANOVA tests for the subscales information and participation in the caregiver sample

Dependent variable	Effect	$\:\mathrm{d}{\mathrm{f}}_{num}$	$\:\mathrm{d}{\mathrm{f}}_{den}$	F	$\:{\eta\:}_{ges}$	*p*
Information	Treat. Setting	1	78	0.00	< 0.001	0.990
	Patients‘ gender	1	78	1.17	0.013	0.282
	Patients‘ diagnosis	2	78	0.17	0.003	0.848
Participation	Treat. Setting	1	88	0.51	< 0.001	0.479
	Patients‘ gender	1	88	0.06	< 0.001	0.806
	Patients‘ diagnosis	2	88	0.18	004	0.840

Treat. Setting = Treatment Setting: inpatient, day hospital; gender: female, male; diagnosis: externalizing, internalizing, neither; $\:\mathrm{d}\mathrm{f}$ = degrees of freedom; $\:{\eta\:}_{ges}$ = explained variance (generalized eta squared)

**Fig. 2:**
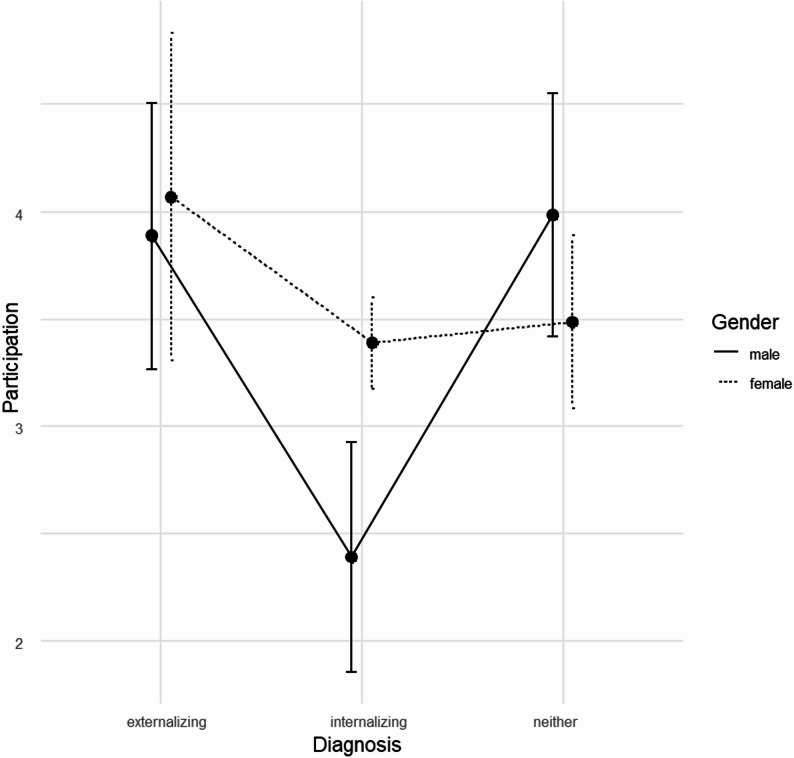
Means and 95% confidence intervals of participation ratings by diagnosis category (x-axis) and gender for the adolescent sample

For the adolescent sample, ANOVA results are somewhat more heterogeneous than for the other samples. Table 8 contains the corresponding test statistics. The rating of participation significantly depended on the type of diagnosis and the interaction of diagnosis and gender. The interaction effect indicates that, while no notable gender differences were observed among adolescents with an externalizing disorder or a neither internalizing nor externalizing disorder, female adolescent patients rated participation distinctly higher than male patients if their diagnosis was classified as internalizing (see Fig. 2). With respect to the ratings of information, adolescents in day hospital gave consistently more favourable ratings.

**Table 8:** ANOVA tests for the subscales information and participation in the adolescent sample

Dependent variable	Effect	$\:\mathrm{d}{\mathrm{f}}_{num}$	$\:\mathrm{d}{\mathrm{f}}_{den}$	F	$\:{\eta\:}_{ges}$	*p*
Information	Treat. Setting	1	131	15.19	0.104	< 0.001
	Gender	1	131	0.40	0.003	0.528
	Diagnosis	2	131	1.97	0.029	0.143
Participation	Treat. Setting	1	129	1.51	0.012	0.222
	Gender	1	129	1.03	0.008	0.313
	Diagnosis	2	129	10.36	0.138	< 0.001
	Gender: Diagnosis	2	129	5.53	0.079	0.005

Treat. Setting = Treatment Setting: inpatient, day hospital; gender: female, male; diagnosis: externalizing, internalizing, neither; $\:\mathrm{d}\mathrm{f}$ = degrees of freedom; $\:{\eta\:}_{ges}$ = explained variance (generalized eta squared)

## Discussion

This study investigated whether the treatment setting (day hospital vs. inpatient treatment) affects the ratings of information and participation of children, adolescents and caregivers. To this end, two new subscales of information and participation were constructed for exploratory purposes. Findings based on these subscales should be interpreted cautiously given their limited reliability and lack of validation. In our sample, participation ratings range from moderate to rather high in both settings by all group samples (mean children: 3.92; caregivers: 4.18; adolescents: 3.41, on a scale from one to five). The setting demonstrates no significant effect on the ratings of participation among children, caregivers or adolescents. This may suggest that the structural and procedural characteristics of inpatient treatment, e.g., a higher number of patients presenting with acute symptoms, 60–70% emergency admissions, shift work within the nursing and socio-educational staff, shorter durations of stay and more, do not preclude participation. This may also suggest that assumptions held by professionals, such as the idea that participation needs more time and effort [40] may be inaccurate [41]. According to a comprehensive systematic review on adolescents’ involvement in mental health treatment, a collaborative relationship between patient and therapist and staff’s capacity are two of the main factors in implementing participation [4]. It seems possible that these aspects can be satisfactorily implemented in both treatment settings despite structural challenges in inpatient wards.

The ratings of information are moderate to positive (mean children: 3.63; caregivers: 4.05; adolescents: 3.46). Adolescents are more satisfied with information provision in the day hospital setting. The subscale information consists of the items about information on medication, coercive measures, post-discharge care and items regarding the discussion of treatment goals with the professionals. Older studies show that patients’ satisfaction with the information received in CAMHS varies depending on the topic. Clearly definable topics, such as information about ward rules, seem to be easier to communicate than information about topics that can vary individually, such as the course of treatment [41]. In our sample, the largest difference in information and participation between the settings exists in the item regarding the joint discussion of treatment goals, especially among adolescents. This result may be associated with the systematic discussion of treatment goals in the adolescent day hospital, which is based on an implemented participatory, patient-orientated treatment plan. This tool is used with the adolescents, but also with the patients’ caregivers. It involves not only doctors and therapists but also the nursing and socio-educational staff. The staff’s assessments and perspectives are discussed with the patients and caregivers. Additionally, this tool is also used to record and discuss symptom changes over time. Interestingly, although caregivers of adolescents in the day hospital are included in the treatment plan, there are no significant differences in the caregivers’ ratings of information between settings. However, these findings should be interpreted with care due to the heterogeneity present in this subsample, which combines caregiver responses related to both children and adolescents, with uneven representation across settings. As for the child sample, we do not know whether there is significant difference between the inpatient ward and day hospital regarding the item “joint discussion of treatment goals”, as the questionnaire for children does not contain these items.

The discussion of treatment goals is an item of the BEST questionnaire and was assigned to the information subscale (see Fig. 1). According to the theoretical model of Wright et al. [3] information and participation are two distinct but related concepts [3]. The discussion of treatment goals is about exchanging information and opinions. The focus is not yet on making a decision, but on gathering and weighing information. The joint discussion of treatment goals can therefore, in line with the model of Wright et al. [3], be seen as a necessary step towards participation. For clinical healthcare teams who are already using the BEST questionnaire on a regular basis and are interested in the aspects of information and participation, the new subscales could shed light on the patients’ and caregivers` perception of information and participation during treatment, so that these important aspects remain in focus. Regularly evaluating and discussing the results within teams could lead to improvements in everyday care. Only the subscales for the child sample, which yielded lowest estimated of internal consistency, may lack precision to be interpreted for individual diagnostics and should be only considered to investigate means trends based on sufficiently large samples.

According to the present results, gender and diagnosis could be associated with the patients’ ratings of participation. However, given the exploratory nature of the analyses, no correction for multiple testing was applied; findings should therefore be interpreted as hypothesis-generating. Younger girls in this sample seemed to rate their possibilities to participate higher than younger boys. Female adolescents who have an internalizing diagnosis seem to feel better included than males with the same diagnosis. This contradicts findings of other studies that demonstrated no significant effect of gender and diagnosis on the perception of participation [7, 8, 14].

### Limitations

Findings based on the subscales should be interpreted cautiously given the limited reliability in some subscales and lack of validation. The observed differences between settings may reflect differences in patient case-mix rather than effects of the treatment setting itself. Due to the observational nature of this study, it does not allow for causal attribution to settings. Most patients who completed the questionnaire stayed longer than two weeks at the hospital; patients who were discharged within less than two weeks are therefore underrepresented. Our calculations did not control for the duration of the treatment. Future research should investigate how patients who only stay short term (less than two weeks) evaluate the information and participation during their stay compared to patients with longer stays. All caregiver findings should be interpreted cautiously due to heterogeneity in the sample, which arises from combining caregivers of child and adolescent cases and an unequal distribution across treatment settings. Due to the small sample size, it was not possible to compare the perception of information and participation of court-committed patients and patients who agreed to their hospital treatment voluntarily. There are hints that patients who are admitted to the hospital by court order may experience less participation than patients who are admitted voluntarily [41]. Other limitations pertain to the child sample, specifically. Formally, there is no minimum age requirement for the BEST questionnaire. The questionnaire is designed as a self-report. Children can receive assistance in completing it. The youngest children in our sample were six years old and required a person to read the questionnaire to them. We do not know whether the BEST-C sufficiently accounts for developmental variability in children younger than 12 years. Additionally, there is no study on the external validity of the BEST version for children because there is no comparable questionnaire in German language for this age group. However, validations of the questionnaires for adolescents and caregivers were good [33]. Future studies with larger sample sizes are needed to examine potential differences. There are hints that patients’ experience of participation can vary over the treatment period [7]. Thus, repeated assessment of patients’ and caregivers’ ratings over the course of the treatment is desirable. The present study did not pursue factor analytic approaches to evaluate the incremental value of the new information and participation scales relative to the established BEST factors. Because the available samples were limited in size and there was a modest amount of missing data, confirmatory factor models could not be estimated reliably. Future research with larger samples should include a comprehensive factorial validation of the new scales.

## Conclusion

The present study examined information and participation in different treatment settings of CAMHS and tried to analyze possible differences between the settings. Only few studies have addressed these differences so far. The present study, using pragmatically developed scales within a routine service evaluation, showed no significant differences for children and caregivers in their ratings of information and participation between the inpatient and the day hospital setting. This may suggest that information and participation could be implemented successfully in both settings despite structural and conceptual differences. However, in the present sample, adolescent patients in a day hospital seemed to be more satisfied with the information provision than adolescents in the inpatient setting. This finding could be caused by confounding variables, such as the case-mix differences between the settings. Another possible explanation could be a participatory patient-orientated treatment planning tool that has been implemented in the day hospital for adolescents. A systematic evaluation of this therapy tool might be helpful to discover its full potential.

## Supplementary Information

Below is the link to the electronic supplementary material.

Supplementary Material 1.

## Data Availability

The dataset analyzed during the current study are available from the corresponding author on reasonable request.

## Footnotes

1 Data of patients who identified with diverse gender identities were excluded from the analyses due to insufficient sample size.
