# Supplementary material for: Perceptions of information and participation in child and adolescent mental health care: a comparison of patient and caregiver reports across inpatient and day hospital settings
Source: Child Adolesc Psychiatry Ment Health. 2026 May 17;20:71. doi: 10.1186/s13034-026-01096-3 (PMC13196158; doi:10.1186/s13034-026-01096-3)
Supplement: Supplementary file 1 — Supplementary Material 1. [file 13034_2026_1096_MOESM1_ESM.docx]

*Table A*

Descriptive statistics and reliability for the original BEST scales and the newly-established subscales participation and information

| Data | (Sub-)Scale | n | #item | Mean | SD | 95% CI | | Cronbach’s Alpha |
| --- | --- | --- | --- | --- | --- | --- | --- | --- |
|  |  |  |  |  |  | Lower | Upper |  |
| Children | BEST-G | 148 | 20 | 3.94 | 0.59 | 3.84 | 4.03 | .85 |
|  | BEST-F1 | 148 | 11 | 4.02 | 0.64 | 3.92 | 4.13 | .77 |
|  | BEST-F2 | 148 | 6 | 3.78 | 0.81 | 3.65 | 3.91 | .77 |
|  | Part | 148 | 3 | 3.94 | 0.79 | 3.81 | 4.06 | .48 |
|  | Info | 118 | 2 | 3.68 | 1.42 | 3.43 | 3.94 | .37 |
| Caregivers | BEST-G | 105 | 22 | 4.06 | 0.80 | 3.9 | 4.21 | .94 |
|  | BEST-F1 | 105 | 11 | 4 | 1.01 | 3.81 | 4.2 | .94 |
|  | BEST-F2 | 105 | 6 | 4.01 | 0.96 | 3.83 | 4.19 | .87 |
|  | BEST-F3 | 105 | 4 | 4.26 | 0.72 | 4.12 | 4.4 | .80 |
|  | Part | 105 | 3 | 4.09 | 1.04 | 3.89 | 4.29 | .79 |
|  | Info | 105 | 4 | 4 | 1.03 | 3.81 | 4.2 | .80 |
| Adolescents | BEST-G | 195 | 27 | 3.3 | 0.07 | 3.2 | 3.4 | .90 |
|  | BEST-F1 | 195 | 10 | 3.49 | 0.83 | 3.37 | 3.61 | .81 |
|  | BEST-F2 | 195 | 9 | 2.92 | 0.93 | 2.79 | 3.05 | .83 |
|  | BEST-F3 | 195 | 5 | 3.62 | 0.88 | 3.49 | 3.74 | .81 |
|  | Part | 195 | 3 | 3.32 | 1.03 | 3.18 | 3.46 | .66 |
|  | Info | 195 | 4 | 3.39 | 0.99 | 3.25 | 3.52 | .67 |

*Notes.* #item: number of item included in each scale; rel: Cronbach’s Alpha reliability; BEST-G: BEST general factor; BEST-F1: therapeutic relationship; BEST-F2: surroundings; BEST-F3: treatment satisfaction; Part: participation; Info: information.
